# Transition from childhood to adulthood in neuromuscular disorders: results from the ERN EURO-NMD survey

**DOI:** 10.1186/s13023-025-04144-x

**Published:** 2025-12-09

**Authors:** Teresinha Evangelista, Houda Ali, Charlotte Handberg, Thomas Sejersen, Ros Quinlivan, Isabella Moroni, Marion Masingue, Susana Quijano-Roy, Antonio Atalaia, Ulrike Schara-Schmidt, Kristl G. Claeys

**Affiliations:** 1 Functional Unit of Neuromuscular Pathology, Department of Neuropathology, Institute of Myology, AP-HP, Pitié-Salpêtrière Hospital, Sorbonne University, Paris, France; 2 Reference Center for Neuromuscular Disorders, Nord/Est/Ile de France, Institut de Myologie, CHU La Pitié-Salpêtrière, APHP, Paris, France; 3 National Rehabilitation Center for Neuromuscular Diseases, Aarhus, Denmark; 4 Department of Public Health, Faculty of Health, Aarhus University, Aarhus, Denmark; 5 Dept of Women´s and Children´s Health, Karolinska Institutet, Stockholm, Sweden; 6 Center for Neuromusculoskeletal Restorative Medicine, Hong Kong Science Park, Shatin, New Territories, Hong Kong China; 7 Centre for Neuromuscular Diseases, UCL Institute of Neurology, London, United Kingdom; 8 Department of Pediatric Neurosciences, Fondazione IRCCS Istituto Neurologico C. Besta, Milan, Italy; 9 Neuromuscular Unit, Pediatric Neurology and ICU department, Raymond Poincaré University Hospital (UVSQ), APHP- Université Paris Saclay, Garches, 92380 France; 10 Department of Pediatric Neurology, Center for Neuromuscular Disorders in Children and Adolescents, University Clinic Essen, University of Duisburg-Essen, Essen, Germany; 11 Department of Neurology, University Hospitals Leuven, Leuven, Belgium; 12 Department of Neurosciences, Laboratory for Muscle Diseases and Neuropathies, KU Leuven, Leuven, Belgium; 13 Functional Unit of Neuromuscular Pathology, Neuropathology Department, Institute of Myology Pitié-Salpêtrière Hospital, APHP, Paris, France

**Keywords:** Neuromuscular diseases, Transition of care, Paediatric neurology, Adult neurology, Rare diseases, ERN EURO-NMD, Continuity of care, Healthcare policy, Care coordinator, Transition programmes, Paediatric to adult care

## Abstract

**Background:**

Neuromuscular diseases (NMDs) are rare, progressive conditions that require lifelong, multidisciplinary care. Advances in diagnosis and treatment have increased survival into adulthood, making the transition from paediatric to adult care a critical stage of its management. However, evidence suggests that transition practices remain inconsistent across Europe. This study aimed to map and evaluate the current transition practices for patients with NMDs across Europe.

**Methods:**

A cross-sectional survey was conducted by the European Reference Network for Rare Neuromuscular Diseases (ERN EURO-NMD) to assess current transition practices across European healthcare providers (HCPs). Sixty-seven healthcare professionals from 20 countries participated. The survey explored training provision, transition structures, professional involvement, timing, psychosocial support, and perceived barriers. Both descriptive and thematic analyses were performed.

**Results:**

Only 29.9% of respondents reported structured transition protocols, and fewer than one in five (17,9%) had received formal training in transition care. The age to initiate transition was reported for most centres at 17–18 years, even though most clinicians identified 15–16 years as the ideal starting point. Multidisciplinary collaboration was present in some centres but was inconsistently implemented. Barriers included insufficient staff, lack of funding, inadequate adult care services, and poor inter-team communication. Post-transfer feedback to paediatric teams was limited. Despite these challenges, 59.7% of respondents believed that ERN EURO-NMD could facilitate improvement in transition care.

**Conclusions:**

Transition care for patients with NMDs in Europe remains fragmented and under-resourced. To ensure continuity and improve outcomes, structured, multidisciplinary transition models are needed. A European roadmap, coordinated by ERN EURO-NMD, could harmonise practices, support professional training, and guide policy development across member states.

**Supplementary Information:**

The online version contains supplementary material available at 10.1186/s13023-025-04144-x.

## Background

Neuromuscular diseases (NMDs) comprise a heterogeneous group of rare conditions primarily affecting the peripheral nervous system. These disorders frequently result in progressive muscle weakness and multisystem complications. Advances in diagnosis and multidisciplinary care have significantly increased life expectancy for individuals with NMDs, emphasising the importance of a structured transition from paediatric to adult healthcare services as a component of long-term management [1].

The transition from paediatric to adult healthcare is particularly difficult for patients afflicted by rare and ultra-rare diseases. Young adults and their carers face a variety of barriers, including lack of knowledge, uncertainty and limited expectations regarding future care and support, as well as financial concerns. The burden of these unique transition issues is further exacerbated by disease-specific challenges and the scarcity of experts among adult healthcare professionals, a commonly reported barrier to a successful transition process [2].

Transition is not merely an administrative transfer but a complex, individualised, and multidisciplinary process that must address the medical, psychological, and social needs of young people with NMDs [3]. Ideally, transition should begin during early adolescence to prepare patients and their families for changes in care models and roles. Effective transition requires coordination among various healthcare professionals, including child and adult neurologists, rehabilitation specialists, psychologists, occupational therapists and social care workers, to ensure continuity and holistic care [4]. Evidence suggests that integrated care models are most effective in enabling seamless transitions between care providers, while transition preparation programmes play a critical role in building patient capacity [2]. Inadequate transition planning can lead to care disruptions, reduced treatment adherence, higher morbidity, and diminished quality of life. The psychosocial challenges of adolescence – such as developing autonomy, navigating education or employment, and adjusting to adult care systems – make structured transition planning even more essential. When implemented effectively, individualized transition programmes can improve patient outcomes, foster empowerment, and reduce healthcare burden [1, 4].

Published evidence suggests that effective transitioning from paediatric to adult care for patients with neuromuscular disorders is associated with improved clinical, psychosocial, and functional outcomes, though this evidence remains limited and mostly qualitative. Reviews show that while transition is increasingly recognized as critical, systematic or high-quality studies quantifying its impact are rare. Consistently, the literature notes that poorly managed transitions can result in negative clinical outcomes, including higher rates of medical complications, increased risk of emergency interventions, breakdown of follow-up, lower adherence to therapy, discontinuity in assistive technology provision, and greater psychosocial distress. Properly planned transitions that address the physical, psychological, educational, and social needs of young people with neuromuscular disorders, and involve families, caregivers, and multidisciplinary teams, are associated with higher patient satisfaction, better adherence, and more stable health status during adulthood [4–7]. 

The aim of this study is to map and evaluate the current transition practices for patients with NMDs across Europe, to identify existing challenges, institutional practices, and areas requiring improvement. The results of this work will inform the development of evidence-based and/or consensus-based recommendations, and it will support the creation of a European roadmap to standardise and optimise the transition of care for patients with NMDs.

## Methods

The ERN EURO-NMD Transition Task Force conducted a cross-sectional survey to assess how the transition from paediatric to adult care is managed in NMDs across Europe. The survey (Supplementary File 1) was developed collaboratively by the ERN Task Force on transition and disseminated via the ERN EURO-NMD internal communication channels to the network’s 82 healthcare providers (HCPs) representatives. Each HCP representative was encouraged to circulate the survey internally to relevant colleagues within their institution. Additionally, we reached out to five NMD UK centres through the British Neurological Society. Because dissemination occurred through institutional and national networks, the exact number of healthcare professionals who received the questionnaire could not be determined, and a precise response rate cannot be calculated. We estimate that more than 100 healthcare professionals from 26 European countries were invited to participate. The survey was open between 26th September and 21st October 2024. The participation was voluntary and anonymous, although respondents were required to indicate their country and HCP. To ensure different perspectives, multiple responses from the same centre were allowed, especially when both paediatric and adult care teams were involved. To address potential variability in interpretation of key terms across countries and specialties, the survey included a glossary of standard definitions. Specifically, ***transition*** was defined as an active, patient-centred process addressing medical, psychosocial, and educational needs as adolescents prepare to move from paediatric to adult healthcare systems. In contrast, ***transfer*** was defined as the discrete event of transitioning care responsibility from one healthcare team to another. The survey contained both closed- and open-ended questions and was structured into eight sections: (1) respondent and institutional demographics; (2) experience with transition; (3) organisational and national frameworks; (4) transition process characteristics; (5) patient and family support; (6) barriers to transition; (7) quality and monitoring of transition care; and (8) future directions and recommendations.

Descriptive statistics were used to summarize categorical variables (frequencies and percentages). Where applicable, chi-square or Fisher’s exact tests were employed to compare categorical groups, and t-tests or Mann-Whitney U tests were used for continuous variables. The threshold for statistical significance was set at *p* < 0.05. Natural Language Processing (NLP) techniques were used to analyse open-ended responses, including thematic coding and keyword extraction, with assistance from ChatGPT for structured content analysis. A country-level inconsistency score was developed to highlight disparities in responses within countries. This score combined the number of “I do not know” responses and the absolute difference between “Yes” and “No” answers to quantify internal variability.

All analyses were conducted in accordance with best practices. No ethical approval was required as the study collected non-identifiable, professional-level opinions and was conducted under the umbrella of an official ERN EURO-NMD initiative.

## Results

***Section. 1: Participants and Institutional Characteristics***

A total of 67 healthcare professionals across 20 European countries participated in the survey, represented 48 identifiable HCPs, as 3 respondents didn’t specify the HCP name. Countries from which no contributions were received included Bulgaria, Ireland, Luxembourg, Norway, Poland and Slovenia.

Respondents represented a range of age groups, with the majority being over 55 years old (*n* = 28; 41.8%), followed by those aged 45–55 years (*n* = 24; 35.8%). A smaller proportion of participants were aged 35–45 years (*n* = 12; 17.9%), while the 25–35 years accounted for only 4.5% of respondents (*n* = 3). Most respondents reported their gender as female (*n* = 40; 59.7%), followed by male (*n* = 25; 37.3%). The majority (*n* = 50; 74.6%) had more than 10 years of clinical experience of working with patients with NMDs. Adult neurologists made up the largest group (*n* = 38; 56.7%), followed by child neurologists (*n* = 26; 38.8%), and other professions including care coordinator, rehabilitation physician, clinical neurophysiologist and clinical researcher (*n* = 3; 4.5%) [8].

**Fig. 1:**
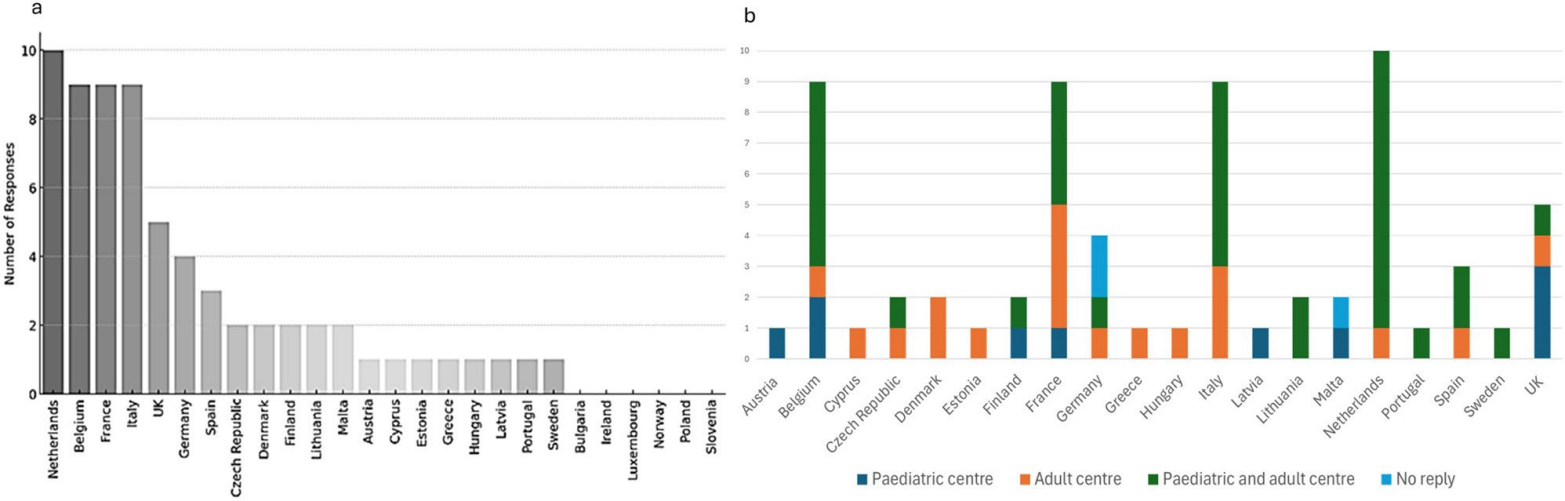
Survey responses by country (**a**) and by country and type of centre (**b**). (**a**) Bar chart representing the number of survey responses per country, with countries sorted in descending order of responses. (**b**) Stacked bar chart showing the distribution of centre types by country

The number of countries involved reflected a broad geographic coverage and provided insights into transition practices across various healthcare systems. It also highlighted the structural diversity within the ERN EURO-NMD network, where some countries have a single affiliated healthcare provider, while others, such as those contributing more responses, have multiple participating centres (Fig. 1.a).

More than 50% (*n* = 35; 52.2%) of respondents worked at centres providing both paediatric and adult care, while the remainder represented either adult-only (*n* = 19; 28,4%)) or paediatric-only (*n* = 10; 14;9%) centres (Fig. 1. b). Three centres (4,5%) did not mention if they were paediatric or adult centres.

***Section. 2: Experience***,*** Training and Preparedness for Transition***

Just over half of participants indicated that they had more than 20 years of experience working with NMD patients (*n* = 34; 50.7%), while 16 (23.9%) had between 10 and 20 years, 13 (19.4%) reported 5–10 years and only 4 respondents (6%) less than 5 years. The estimated mean duration of clinical experience was 17.9 years (SD = 8) with a median of 25 years. This reflects a highly experienced respondent group, with the majority having long-standing involvement in NMD care.

Despite extensive clinical experience, only 17.9% (*n* = 12/67) of respondents had received formal training in transition care, with younger professionals (< 35 years; *n* = 3) reporting no exposure to such training. The majority (*n* = 55; 82.1%) had no specific training, often relying on experience or informal mentorship.

Furthermore, 46 participants (71.6%) provided free-text comments regarding training on transition. The key themes identified in the free-text analysis were lack of formal training, experience-based learning and the importance of multidisciplinary collaboration as an alternative to structured education. Several participants described transition as a process that occurs organically within institutional culture, often guided by mentorship, literature, or local expertise. Multidisciplinary collaboration was frequently cited as a key facilitator functioning as an informal learning environment. Interestingly, some UK centres reported a structured approach making use of the “Ready Steady Go” programme [9] and the presence of designated transition nurses.

We further investigated whether participants felt adequately prepared to support patients with NMDs during the transition period. In the survey, 67.2% (*n* = 45) of respondents reported feeling adequately prepared while 32.8% (*n* = 22) indicated that they do not. To explore potential factors associated with perceived preparedness, a Pearson correlation analysis was performed to assess the relationship between feeling adequately prepared and several demographic and professional variables: age range, years of experience, specific training on transition, and medical specialty. However, none of these associations were statistically significant (p-values > 0.05). A total of 50 participants (74,6%) provided free-text responses, of these 4 were not included in the free-text analysis as they did not address the question. Most responses came from professionals with over 20 years of experience (*n* = 25; 54.3%), followed by those with 10–20 years (*n* = 9; 19.6%), 5–10 years (*n* = 9; 19.6%), and less than 5 years (*n* = 3; 0.1%). Most respondents (*n* = 38; 82.6%) had not received specific training. Analysis of the qualitative responses suggests that training increased confidence and provided structure for managing transition. On the other hand, the lack of training contributed to uncertainty and reliance on personal or institutional experience. However, among professionals who indicated a lack of formal training (*n* = 38), many reported feeling adequately prepared (*n* = 24/38; 63.2%), often citing extensive clinical experience as a compensatory factor.

**Fig. 2:**
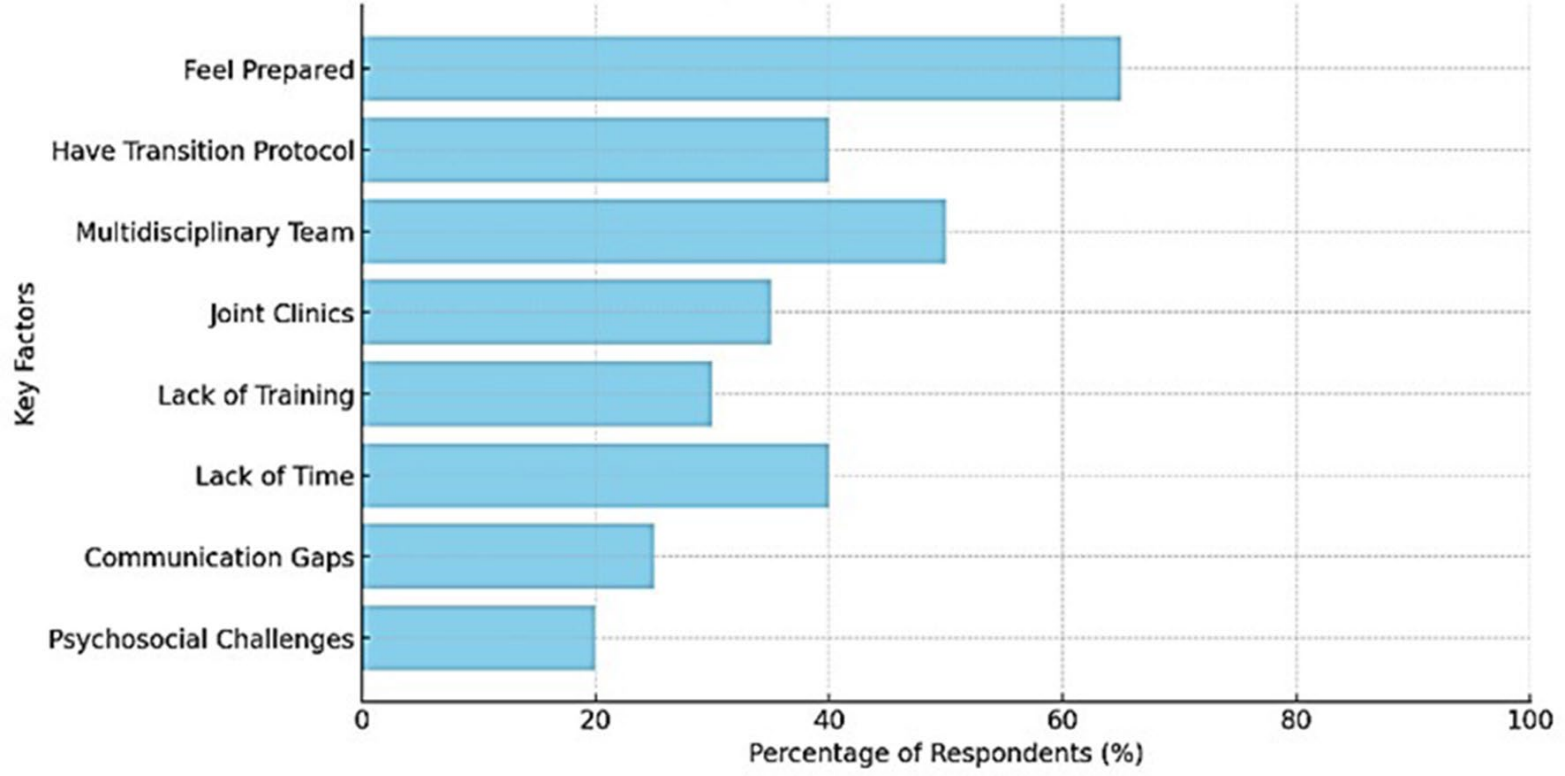
Factors influencing preparedness for transition to adult care. Bar chart illustrating the proportion of respondents identifying specific factors influencing preparedness for transition to adult care. Percentages represent the proportion of respondents selecting each factor

The key concepts that emerged from the free text analysis were confidence through experience versus lack of training. The three main factors influencing preparedness for transition to adult care were fragmentation of care, multidisciplinary teams’ roles, and the psychological and social readiness of the patients **(**Fig. 2**).**

Even experienced professionals acknowledged that formal training and protocols would enhance their ability to manage transitions. Those with structured joint care models reported feeling more confident and supported. An important aspect mentioned is the psychosocial support for patients, which is often overlooked, and many professionals complain about the lack of resources to assist patients with the emotional and social aspects of the transition. Free-text comparisons between trained and untrained participants revealed some differences regarding preparedness. Common themes in trained staff responses were structured processes, multidisciplinary collaboration, and greater confidence. *The following sentence exemplifies that sentiment: “The training provided a structured framework*,* making the transition smoother for both patients and staff.”* Untrained professionals frequently described uncertainty, lack of resources, and reliance on experience. *Example: “We do our best*,* but there is no formal training or guidance on how to handle these transitions effectively.”*

***Section. 3: Structure and Organisation of Transition Care***

In this section, we evaluated how the transition of care process, either in general or specifically for NMDs, is organised at both the HCP and the national level.

Only 29.9% of respondents (*n* = 20) reported the presence of general transition protocols in their institution, and a similar proportion reported the existence of NMD-specific protocols (*n* = 19; 28.4%). Lack of awareness was common, with 26.9% (*n* = 18) and 16.4% of respondents (*n* = 11), respectively, being unaware of whether such protocols existed.

We have also assessed the availability of national-level transition frameworks. About half of respondents ignored whether such programmes existed in their country (*n* = 34; 50.7%). On the other hand, 17 respondents (25.4%) indicated that no national transition programmes or guidelines were in place, and only 16 respondents (23.9%) confirmed the existence of national transition programmes. The large number of “I do not know” responses underscores the need for more effective dissemination of existing policies. When asked about the availability of NMD-specific transition programmes at the national level, 34 respondents (50.7%) reported that their country does not have any transition programs specifically for NMDs in place, 25 (37.3%) were unsure, and only 8 (11.9%) confirmed the existence of such programmes.

***Section. 4: Characterisation of the transition process***

We assessed both the actual age at which patients are prepared for transition to adult services, as well as the age range that respondents consider ideal for initiating the process. Respondents indicated their choices for each question using four predefined categories: ≤12, 13–14, 15–16, and 17–18 years old.

Most respondents reported initiating transition planning at 17–18 years (*n* = 29; 43.3%), followed by 15–16 years (*n* = 24; 35.8%), 13–14 years (*n* = 11; 16.4%), and ≤ 12 years (*n* = 3; 4.5%). When asked about the ideal age to begin transition, most selected 15–16 years (*n* = 32; 47.8%) as the preferred age range, followed by 13–14 years (*n* = 21; 31.3%), 17–18 years (*n* = 10; 14.9%), and ≤ 12 years coming last (*n* = 4; 6.0%). These findings reveal a clear mismatch between current practice and perceived best practice, with earlier transition planning preferred across specialties and countries (Supplementary file 2).

**Fig. 3:**
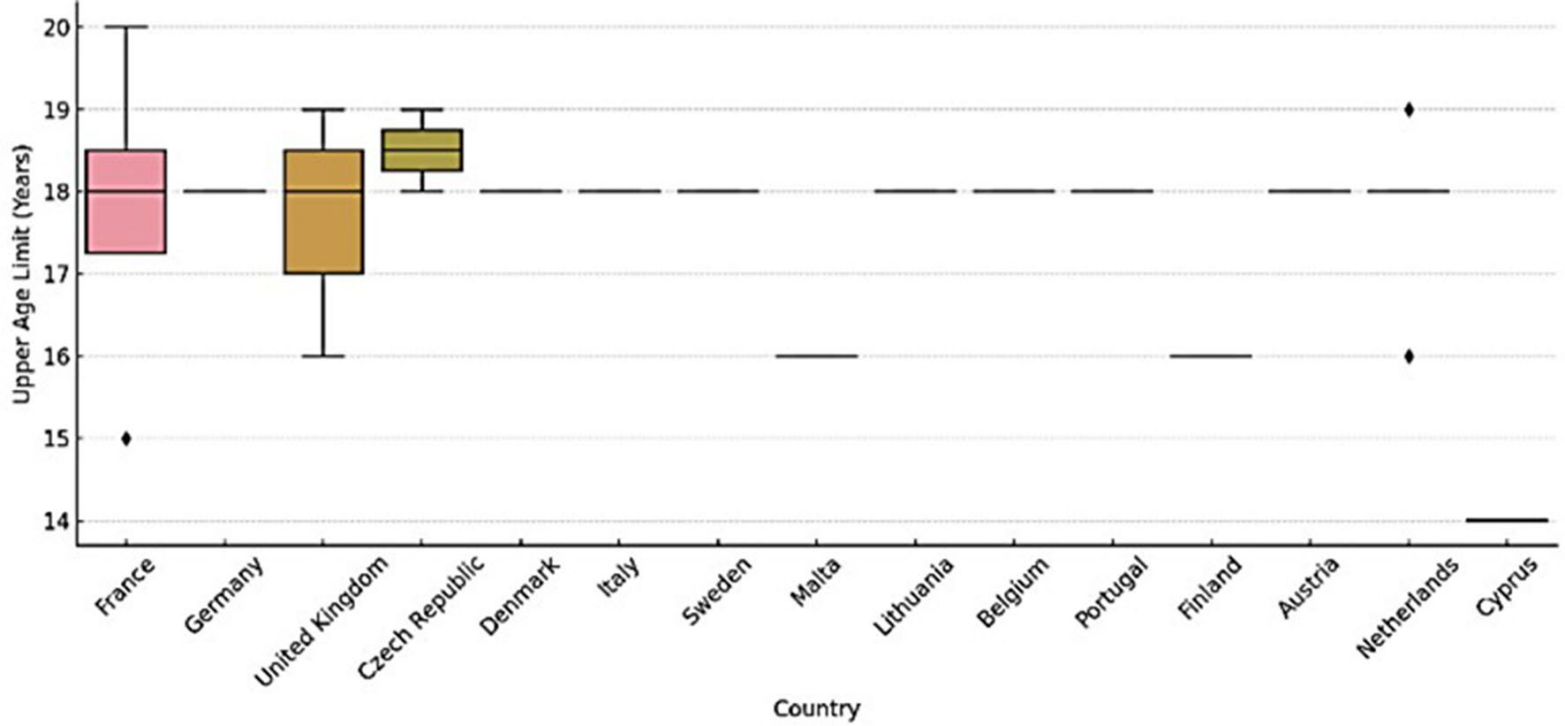
Reported upper age limits for transition to adult care by country. Boxplot illustrating the distribution of upper age limits for transition to adult care as reported by healthcare professionals in each country. Each box represents the interquartile range (IQR), with the horizontal line indicating the median. Whiskers extend to the most extreme data points within 1.5 × IQR, and dots indicate outliers. The figure highlights both between-country variability and within-country differences, with some countries showing a consistent limit of 18 years and others reporting broader ranges

Institutional age limits for transfer were reported by 34 respondents (50.7%), most set at 18 years, though flexibility up to 21–22 years was noted in Austria, Germany and Italy, where child neurologists may continue care based on clinical or psychosocial maturity (Fig. 3).

Regarding the organisation of the transition, most respondents (*n* = 42; 62.7%) reported a structured process, while 29.9% (*n* = 20) indicated a one-time transfer and 7.5% (*n* = 5) were unsure, suggesting that structured transition planning predominates but is not yet universal (Supplementary file 2).

**Fig. 4:**
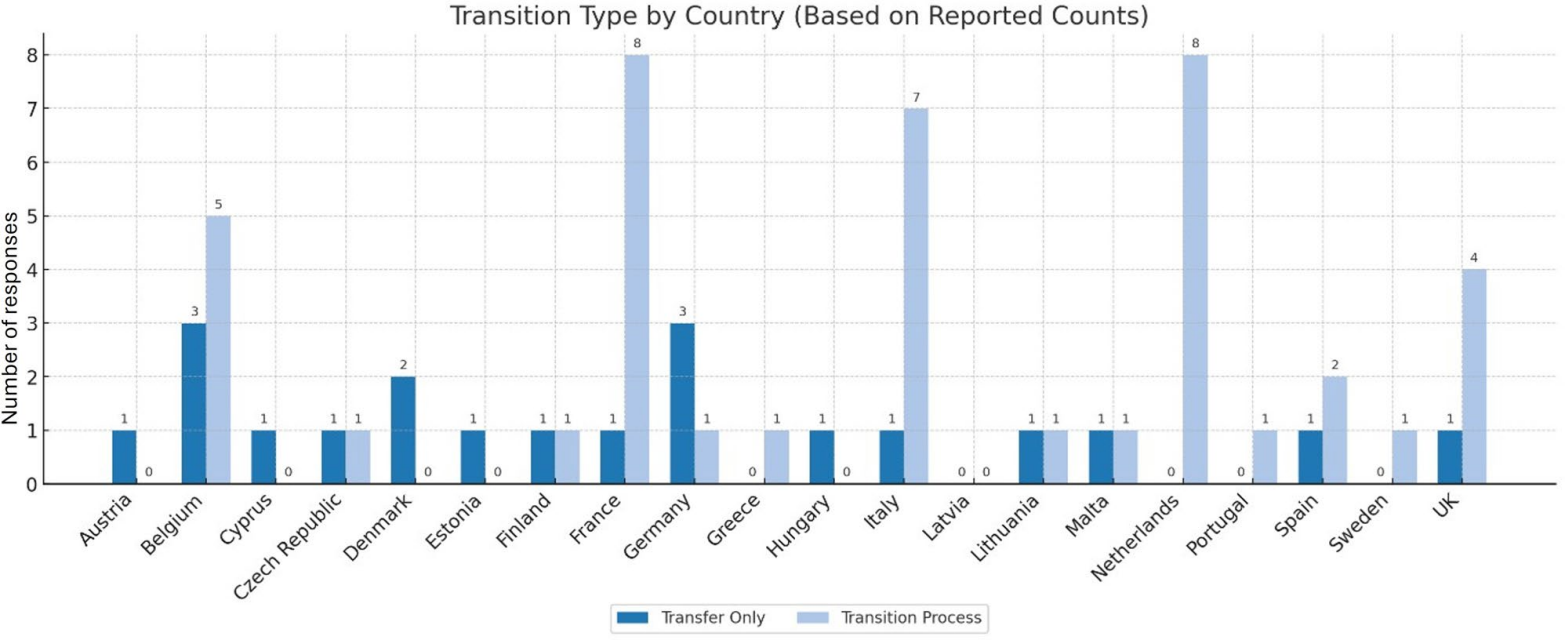
Reported transition models by country. Bar chart showing the number of centres in each country reporting either a “Transfer Only” approach (dark blue) or a “Transition Process” (light blue). Data are based on aggregated responses from individual centres. The figure highlights variability in transition practices across Europe, reflecting differences in how care for patients with NMDs is transferred from paediatric to adult services

We explored the coordination between paediatric and adult teams. Regular joint meetings between paediatric and adult teams were reported by 41 respondents (61.2%), while 26 respondents (38.8%) indicated that no such meetings take place. Practices vary widely, even within the same country. For instance, in Belgium, five respondents indicated that these meetings are held while four don’t. Similar differences were seen in Italy, France, the Netherlands, and the UK. These variations reflect local practice differences and point to a lack of national standardisation (Fig. 4).

Among the 41 respondents who reported regular meetings or joint appointments at their centre, 16 (39%) indicated that these took place in paediatric clinics, 9 (22%) in adult clinics, and 16 (39%) in both settings. Additionally, 27 respondents (65.9%) stated that both paediatric and adult professionals are involved with similar frequency in these meetings. According to 12/41 respondents (29.3%), paediatricians predominated, and only one respondent reported adult-led meetings. One did not provide a response to this question.

### Interdisciplinary coordination and continuity of care

When enquired about the availability of a preparatory transition programme prior to the first consultation in the adult clinic, most respondents (*n* = 41; 61.2%) reported that no such programme exists. Only 18 respondents (26.9%) indicated having a preparatory programme in place, while 8 (11.9%) answered “I do not know”. Among those with a programme, 7 respondents (38.9%) reported that it is coordinated jointly by paediatric and adult healthcare professionals, 5 (27.8%) by paediatricians alone, two (11.1%) by adult neurologists, and four (22.2%) by other professionals such as specialist nurses, care coordinators, neuromuscular advisors, or pulmonologists.

We collected free text comments on the content of the preparatory transition programme where it exists (*n* = 18) and received 17 answers. The qualitative content analysis revealed five key domains: (1) Information Sharing, with almost all respondents emphasising the importance of providing information – both medical and educational – to patients and families. The common elements of this information sharing were medical history transfer, transition guides or flyers, and family education. (2) Structured Tools: some centres utilise formal programs or standardised tools (SOP and transition databases) to guide the transition process. (3) Multidisciplinary and Interdisciplinary Involvement: several centres highlight the role of the multidisciplinary team or cross-specialist communication. (4) Patient-Centred Engagement and Readiness Assessment: some responses highlight efforts to engage young people and assess their understanding/readiness actively. (5) Continuity of Care and Joint Consultations: some centres provide joint appointments or ensure care continuity.

Respondents were then asked to select the tools used in their practice to assess transition readiness, among the following options: Transition Readiness Assessment Questionnaire (TRAQ) 5.0; the California Healthy and Ready to Work (HRTW) transition assessment tool; the Self-Management Skills Assessment Guide (SMSAG); the TRxANSITION Scale; Other; or None. Most responses (*n* = 52; 77.6%) indicate “None”. TRAQ 5.0 and SMSAG were selected by two respondents each (3.0%), and only one respondent indicated using the TRxANSITION Scale (1.5%). Eleven participants (16.4%) selected “Other”, with textual comments indicating that they mainly rely on subjective clinical assessment, impressions, or individual discussion with patients rather than standardised tools.

We inquired about who is responsible for advising parents/young adults about the transition process. The possible answers to the question were predefined as: A designated transition coordinator; The patient’s paediatric neurologist; The patient’s general practitioner; Other. The patient’s paediatric neurologist was identified as the primary professional responsible for transition guidance (*n* = 57; 85.1%). Only five respondents (7.5%) mentioned a designated transition coordinator, and five selected “Other”, mainly from the Netherlands and the UK, with comments mentioning multidisciplinary team members and specialist nurses.

To investigate how continuity of care is maintained during transition, we asked respondents to identify the mechanisms in place (with multiple responses allowed). The most commonly reported were shared electronic records (*n* = 52; 77.6%), followed by secure transfer of relevant medical records (*n* = 32; 47.8%), supervision by a care coordinator or case manager (*n* = 17; 25.4%), and two respondents indicated using standardised protocols and check lists (3.0%). Twelve respondents selected “Other” and provided free-text responses showing that many centres have shared care models, where the same clinicians (or team members) treat the patient in both paediatric and adult settings. Others mention regular case discussions between paediatric and adult teams and comprehensive transition letters prepared by the paediatric team. One comment mentioned patient-delivered records as a practical tool.

***Section. 5: Patient and Family Support***

Psychologists (*n* = 37; 55.2%), social workers (*n* = 26; 38.8%), and transition nurses (*n* = 19; 28.4%) emerged as the main professionals addressing the psychosocial needs of patients and families. Youth workers were rarely involved (*n* = 2; 3.0%). An additional 19 respondents (28.4%) selected “Other”, indicating the presence of alternative or informal support roles. The qualitative analysis of the open-text responses revealed four major themes related to how psychosocial support is managed: (1) Clinician-Led Support: in several centres, paediatric and adult neurologists address psychosocial needs directly, in the absence of dedicated professionals. (2) Specialised Nurses or Nurse Practitioners: in countries such as the Netherlands and the Czech Republic, specialised NMD nurses or care coordinators were described as central figures in providing psychosocial support. (3) Gaps in Formal Psychosocial Teams: several centres explicitly reported the absence of structured psychosocial services. Support in these settings was often provided on a case-by-case basis, reflecting variability in staffing and institutional resources. (4) Mental Health Professionals: a small number of centres reported access to psychiatrists or family therapists as part of the transition support framework.

***Section. 6: Barriers to Transition***

### Overview and scoring methodology

To identify perceived barriers in transitioning patients with NMDs, respondents (*n* = 67) were asked to rate their level of agreement with 10 predefined challenges. Ratings were provided using a 5-point Likert scale: Strongly Agree, Agree, Neutral, Disagree, and Strongly Disagree. To quantify and compare perceptions, a scoring system was applied: Strongly agree = 4 points; Agree = 2 points; Neutral = 0 points; Disagree = -2 points; Strongly disagree = -4 points. The mean score for each item was calculated both globally and by country. A higher score indicates a greater perceived barrier.

### Global rankings of transition barriers

The highest-ranking barriers across all countries were the need for a multidisciplinary team (mean score: 1.9), lack of education and training in transition (1.7), and insufficient financial support for transition programmes (1.6). These were followed by the lack of adequate clinical settings to receive young adults with complex needs (1.3) and caregiver preference to remain in paediatric care (0.8). The lowest-scoring barriers included limited adult providers willing to accept patients (-0.3) and difficulty obtaining paediatric records (-2.2).

### Country-level variability

Heatmap analysis of average scores by country (Table 1) revealed significant variation in how different countries perceive and prioritise barriers to transition. For example, countries like Belgium, the Czech Republic, and Germany frequently reported high scores for structural barriers, while others, such as Cyprus, Latvia, and Portugal, displayed more mixed perceptions.

The need for a multidisciplinary team was rated highly across nearly all countries, including maximum scores in Austria, the Czech Republic, France, Germany, Greece, Hungary, and Italy. Similarly, lack of education/training and financial support were consistently perceived as barriers, with multiple countries assigning scores > 2.0.

Some countries exhibited strong internal consistency (e.g., Germany, France) while others showed more heterogeneous patterns, possibly reflecting differences in regional healthcare organisation or institutional policies.

**Table 1:**
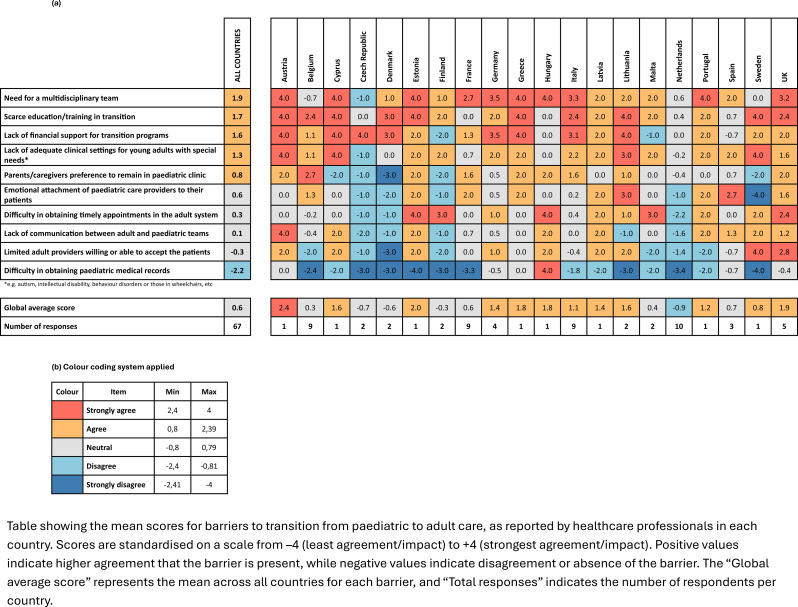
Mean scores for reported barriers to transition by country

Additional barriers were identified through thematic analysis of open-text answers provided by 25 healthcare professionals from 10 countries. Five overarching themes were identified: 1 - Time and resources constraints; 2- Structural and System level gaps like fragmentation between paediatric and adult care and the loss of support services in adult care; 3-Legal, Policy, and Organizational Issues like the absence of legal frameworks 4 - Coordination and Communication Barriers between paediatric and adult teams; 5 - Attitudinal and Cultural Barriers. The qualitative data reinforced many of the previously ranked barriers but also revealed less tangible and often overlooked challenges such as logistical coordination, legal gaps, and cultural resistance. (Supplementary file 2)

### Financial barriers to transition

In this section the participants were asked about possible financial barriers specifically associated with transitioning. More than half (*n* = 37; 55.2%) of respondents described financial obstacles imposed by health authorities. Common concerns included age-based cut-offs in funding and loss of supportive services after the age of 18 (e.g., physiotherapy, psychological support), fragmented reimbursement for multidisciplinary care, and lack of dedicated budgets for transition-related roles. On the other hand, 25 respondents (37.3%) did not report financial barriers, two (3.0%) were not aware of any, another two (3.0%) did not know, and one participant (1.5%) did not respond. (Supplementary file 2)

***Section. 7: Quality Assessment of Transition***

To explore how the quality of transition care is evaluated, respondents were asked whether their institution has mechanisms in place for this purpose. Only seven out of 67 respondents (10.4%) reported the existence of such mechanisms, while 52 respondents (77.6%) indicated that none are in place, and eight (11.9%) were unsure. When analysed by region, the lack of quality monitoring mechanisms was consistently observed across Europe.

Six out of the seven respondents that answered positively provided more detailed answers: three (France, Italy, UK) referred to the use of structured or semi-structured questionnaires, including annual assessments linked to rare disease centres or ongoing validation of dedicated instruments. Two respondents (Netherlands, UK) mentioned the use of patient feedback forms, satisfaction audits, or focus groups as tools for quality monitoring. One respondent in Germany described relying on informal communication with patients and families rather than standardised measures.

The use of specific metrics or indicators to evaluate the effectiveness of the transition process was reported by 4 out of 67 respondents (6.0%). The affirmative responses were distributed across a small number of countries, including the Czech Republic, Italy, Portugal, and the UK.

Respondents were asked whether the paediatrician or paediatric neurology team receives any follow-up information about the patient visits in the adult clinic during the first year after transition. Only 14 respondents (20.9%) reported that such information is always provided, 16 (23.9%) stated it is often shared, and 24 (35.8%) indicated it is communicated occasionally. In contrast, 8 respondents (11.9%) reported that such follow-up never occurs, and 5 (7.5%) stated they did not know. These results suggest that while follow-up communication between adult and paediatric teams is common, it is not systematic across centres.

***Section. 8: Future Directions and ERN EURO-NMD Role***

With this section, we aimed to explore suggested improvements to the transition process for patients with NMDs, as proposed by healthcare professionals across Europe. In addition, we sought to understand the perceived role of the European Reference Network for Rare Neuromuscular Diseases (ERN EURO-NMD) in driving and supporting these changes.

### Qualitative insights on proposed improvements

A total of 48 free-text responses were received, and as many respondents addressed multiple issues, the resulting themes are overlapping and not mutually exclusive.

A strong emphasis was placed on the importance of joint clinics between paediatric and adult teams, and the integration in the multidisciplinary team of psychologists, social workers, and educators (≥ 15 responses), underscoring the need for holistic care during transition. Respondents very frequently emphasised the need for increased resources and funding, including adequate staffing, infrastructure, and financial support to enable joint consultations and multidisciplinary meetings.

One frequently cited recommendation (≥ 12 responses) was the need for dedicated roles and coordinators to oversee and structure the transition. Respondents called for the introduction of transition-specific personnel, such as transition coordinators, case managers, or transition nurses, to ensure continuity and organisation throughout the process.

Several healthcare professionals (≥ 10 responses) highlighted the lack of clear protocols, structured pathways, or national guidelines for managing transition. Themes related to education, preparation, and family involvement (≥ 7 responses) also emerged, with the respondents stressing the importance of early transition planning, patient and caregiver education and active participation of family doctors.

**Fig. 5:**
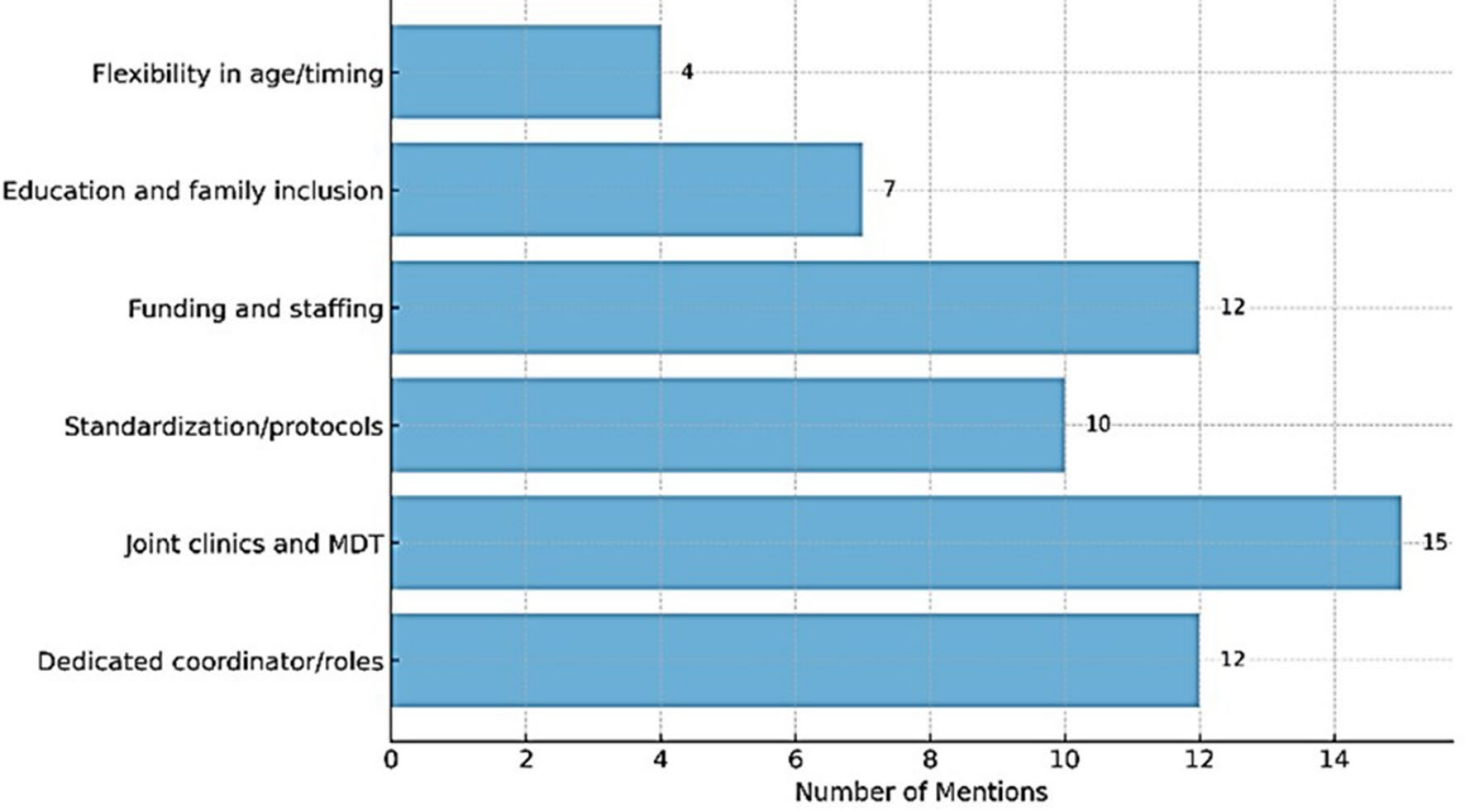
Key Themes: Suggested Improvements for Transition in NMD Care (*n* = 48). This bar chart illustrates the frequency with which specific themes were mentioned as suggested improvements for the transition process in neuromuscular disease (NMD) care, based on responses from 48 participants. The x-axis represents the *Number of Mentions*, and the y-axis lists the identified themes. Bars are scaled proportionally to the number of times each theme was cited

Finally, concerns were raised about the rigidity of age-based transition criteria (4 responses). Several respondents advocated for a more flexible, individualised approach to transfer, as opposed to adhering strictly to the age of 18. One example comes from the Czech Republic, where a respondent recommended *“looser rules on age by insurance companies*,*”* reflecting a broader call for adaptable policies that reflect patient needs rather than arbitrary age thresholds (Fig. 5).

A cross-country analysis revealed both shared priorities and context-specific challenges in improving transition. Resource-related barriers (including staffing, space, and funding) were reported across all regions. Respondents from Italy and the Netherlands frequently emphasised the importance of joint paediatric-adult care and psychosocial support, while several Eastern and Southern European countries called for increased national attention, policy development, and training.

### Quantifying transition priorities: cross-country needs assessment of key measures

The 67 respondents were asked to evaluate the perceived necessity of eight specific measures aimed at improving the transition process. The proposed measures included: (A) A transition protocol, (B) Including the patient’s views and preferences in the planning of transition; (C) A dedicated coordinator responsible for transition; (D) Implementation of national recommendation about adolescent’s transition to adult care system; (E) Joint consultations between adult and paediatric teams; (F) Obtaining financial support; (G) Introducing transition courses in the training programmes for child and adult neurologists; (H) Establishment of a network of specialized care, including physicians, who care for adolescent and young adults with NMDs.

Each item was rated on a 5-point Likert scale, where 1 indicated a *minor need* and 5 indicated a *maximum need*. To aid interpretation, both global average scores per item and country-specific averages were calculated. Higher scores reflect greater perceived importance of the corresponding measure. For cross-country comparisons, the results were color-coded using predefined score intervals: scores between 4.01 and 5.00 were categorized as *Maximum need*, 3.01–4.00 as *High need*, 2.01–3.00 as *Moderate need*, and 1.00–2.00 as *Minor need* (Table 2 (a and b).

**Table 2:**
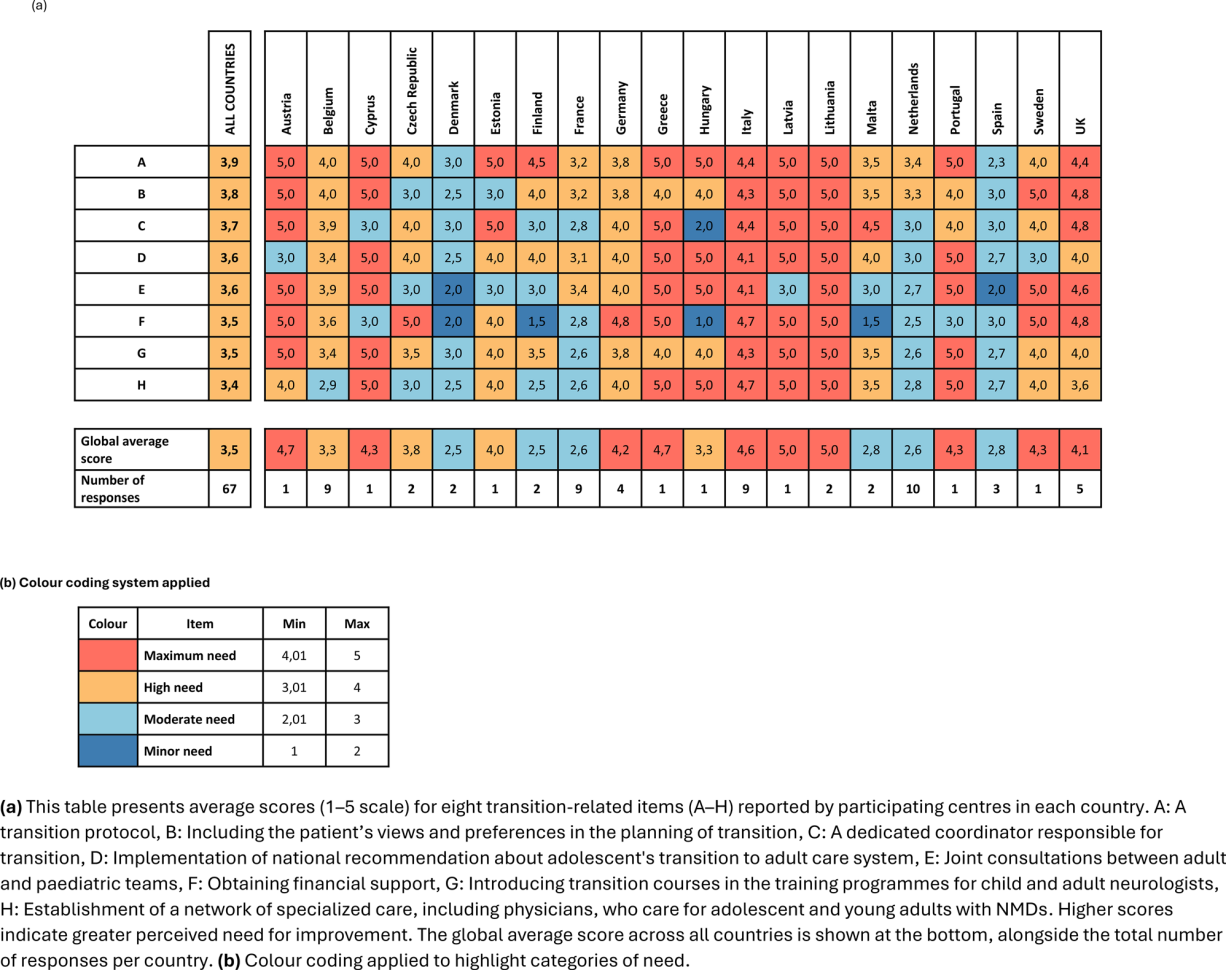
**(a and b)** average need scores for transition in neuromuscular disease care across European countries

The analysis shows that all proposed items scored in the “High need” category globally (mean scores between 3.01 and 4.00), suggesting consensus that all proposed actions are important. The top three priorities across countries were: (A) A transition protocol (Mean = 3.9), (B) Including the patient’s views and preferences in transition planning (Mean = 3.8) and (C) A dedicated coordinator responsible for transition (Mean = 3.7). Other measures, including national recommendations, joint consultations, financial support, and transition-specific training, also received high ratings. The lowest-scoring item – development of a specialized care network – still fell within the *High need* range (Mean score = 3.4).

Country-level variations were notable. While Italy, Lithuania, and Malta reported consistently high scores (up to 5.0) across most items, countries like Denmark, Estonia, and Finland reported lower average scores (ranging from 2.5 to 2.8), particularly for items such as financial support and specialized care networks. The United Kingdom, France, Greece, and Hungary showed strong alignment with the global trend. Austria and Cyprus, each with only a single respondent, scored all items at the maximum value (5.0), which should be interpreted with caution due to limited representation. Certain items showed greater variability in perceived importance across countries. For example, “obtaining financial support” and “introducing transition-related training for clinicians” had the widest range of scores, reflecting differing national priorities or existing support infrastructures.

### Perceived role of European reference networks in optimising transition care

Participants were asked whether European Reference Networks - specifically EURO-NMD - could play a role in optimising the transition process for patients with NMDs. Among 67 respondents, 40 (59.7%) indicated “Yes,” 6 (9.0%) responded “No,” and 21 (31.3%) selected “I do not know”. Full consensus on the positive role of EURO-NMD was observed in several countries, including Germany, Hungary, Cyprus, Estonia, and Sweden, where all respondents answered affirmatively. Italy (8/9 responses; 88.9%) and the Netherlands (6/10 responses; 60%) also reported high levels of agreement. In contrast, greater levels of uncertainty and scepticism were noted in countries such as France, where 3/9 respondents (33.3%) answered “No” and 2/9 (22.2%) were unsure. These findings suggest a generally positive perception of EURO-NMD’s potential to support improved transition care, while also highlighting variable levels of awareness, engagement, or perceived relevance across different national contexts.

The 40 respondents who answered “Yes” to the question about the role of EURO-NMD were invited to elaborate on how the network could contribute to optimising transition care. A total of 39 free-text comments were received. Thematic analysis revealed six major categories, as summarised in Table 3. Overall, respondents envisioned EURO-NMD as a key facilitator in areas such as standardisation of practices, education, and policy advocacy. There was strong support for the development and dissemination of transition guidelines, toolkits, and the promotion of cross-country sharing of best practices.

**Table 3:** Themes and frequency from the free text analysis of suggestions for improving transition in neuromuscular disease care at the European level

Theme	Frequency (mentions)	Example Quotes
Developing European Guidelines and Protocols	23 (58.9%)	“Provide general guidelines and indication of the tools to be used” (Italy)
Training and Education (clinicians, patients)	13 (33.3%)	“EURO-NMD webinar… training for patients and clinicians” (Netherlands)
Advocacy and Political Pressure	8 (20.5%)	“Raise political awareness” (Germany), “pushing authorities” (Italy)
Sharing Best Practices and Models	6 (15.4%)	“Exchange of effective protocols to adapt locally” (Netherlands)
Establishing a Standard Model or Template	5 (12.8%)	“Template for transfer process” (Netherlands), “generalizable model” (Malta)
Patient Engagement and Needs Mapping	2 (3.4%)	“Input from transitioned patients” (France)

This table summarizes themes identified on how to enhance the transition process for patients with neuromuscular diseases across Europe. Each theme is listed with its frequency of mentions (percentage of respondents) and example quotes illustrating the suggested action

### Bottlenecks in optimising transition care: perceived barriers across Europe

When asked about bottlenecks in the optimisation of transition care, the qualitative analysis (*n* = 43/67; 64,2%) showed that the most reported barriers included once again lack of financial resources (60% of responses), insufficient staffing, and time constraints. Many respondents also identified systemic challenges such as fragmented healthcare structures, administrative hurdles, and a lack of political or institutional prioritisation of transition services. A regional analysis revealed distinct patterns. Respondents from Southern Europe most frequently cited multiple simultaneous bottlenecks, particularly those related to financial constraints, healthcare workforce limitations, and policy fragmentation. In Western Europe, common themes included funding, time pressure, and the need for clearer protocols. Central European countries highlighted systemic barriers and the absence of integrated care pathways. Baltic and Nordic regions emphasised time and personnel shortages, while Eastern Europe pointed to cultural and behavioural challenges among healthcare providers and families as a barrier to effective transition. These findings suggest that while certain barriers are universal, such as resource limitations and time constraints, others are more context-specific, reflecting underlying differences in healthcare infrastructure, national policies, and professional culture.

### Key facilitators of transition optimisation: roles for European, National, and local stakeholders

The last question asked who could facilitate optimisation of transition care. 40 open-text answers were collected, with respondents most frequently citing national health systems (52.5%) and European bodies or networks such as EURO-NMD (47.5%). Many highlighted the importance of combined actions across European and national levels, with roles also identified for local hospital management, scientific societies, and patient advocacy groups. Suggested mechanisms included the development of standardised protocols, coordinated funding, and structured training for multidisciplinary teams.

A regional comparison revealed important differences in emphasis. Respondents from Southern Europe most strongly advocated for both European and national engagement, along with the development of shared protocols and involvement of patient advocacy groups and scientific societies. Western European countries also emphasised national health systems but placed greater attention on local institutional leadership and funding allocation at the hospital level. In contrast, responses from Central Europe and the Nordic/Baltic regions focused more on the role of national systems and European guidance, with less mention of civil society or local institutional support.

## Discussion

This survey reveals significant variability and fragmentation in the transition process from paediatric to adult care for patients with neuromuscular disorders (NMDs). Despite the growing awareness of transition as a critical component of lifelong care in rare diseases, our findings highlight persistent structural, educational, and organisational gaps in how this process is implemented across Europe.

A key finding is the mismatch between perceived best practices and actual implementation. While most respondents acknowledged that transition should begin by 15–16 years of age, in practice, transition planning typically starts later – often only at 17–18 years or at the point of transfer. This delay may reduce opportunities for gradual preparation and psychosocial support, both of which are considered essential to successful outcomes [5, 10].

The absence of structured training emerged as a major issue, independently of the age range of the respondents or geographic location. Although most respondents were experienced clinicians, only a minority had received formal education or guidance in transition care. Most clinicians acquired their knowledge through clinical experience or informal mentorship. Younger professionals were particularly underexposed, raising concerns about future capacity to deliver coordinated care. While our data suggest that experience may partly compensate for the lack of formal training, the reverse could also be true—that structured training might enhance preparedness among less experienced professionals. However, this relationship could not be formally assessed in our study and warrants further investigation. The lack of standardised educational pathways mirrors broader inconsistencies in institutional and national protocols [7, 11]. In many countries, even within the same centre, respondents reported uncertainty about the existence of transition frameworks or policies. This suggests an urgent need for more transparent communication, documentation, and training within multidisciplinary teams, as the various practitioners’ s confidence in their ability to support young patients throughout transition appears to play a critical role in the successful implementation of this process.

Most centres lacked dedicated professionals to address psychosocial needs, leaving neurologists or general practitioners to provide informal support.

Our survey shows that very few centres monitor transition quality. Only 10.4% of respondents indicated the existence of formal mechanisms to assess the quality of transition processes. Among these, tools were generally limited to satisfaction surveys or informal meetings. Only 6% of respondents reported the use of performance metrics or outcome indicators. Regional disparities were evident, with Western European centres more likely to engage in quality monitoring than their Eastern or Southern counterparts. The virtual absence of standardised indicators for transition quality presents a critical gap. The development of simple, validated outcome measures – such as readiness assessments, post-transition retention, or patient-reported experience metrics – is urgently needed to track progress [12, 13].

Fragmentation of care continues to hinder effective transition. Although joint meetings and interdisciplinary collaboration were reported by over half of the respondents, this was not consistently implemented across centres. Moreover, communication after transfer was often informal or absent, particularly in adult-led systems where feedback loops to paediatric teams are not systematically maintained. This lack of continuity can affect treatment adherence, disease monitoring, and long-term outcomes [1, 3].

Financial and systemic barriers were also prevalent. Respondents across several regions highlighted the loss of services post-transfer – especially physiotherapy, psychological care, and social support – due to age-related funding limitations. The absence of dedicated reimbursement mechanisms for transition activities (e.g., case managers, multidisciplinary clinics) further exacerbates inequality in care provision.

While many of these aspects – such as access to psychological support, financial coverage, or multidisciplinary coordination – are not unique to the transition phase and remain essential throughout the lifespan of patients with NMDs, they often become particularly critical at the transition interface. The discontinuity or loss of these services during transfer from paediatric to adult systems was frequently perceived as a major barrier to successful transition. Addressing these issues would therefore not only improve the immediate transition experience but also strengthen the quality and sustainability of adult care that follows.

The prevalence of resource-related and structural barriers in Southern and Eastern Europe echoes long-standing North-South disparities in healthcare financing and service delivery [14]. Addressing these inequities is essential to achieving transition care models that are both effective and equitable.

Despite these challenges, most healthcare professionals surveyed believed that EURO-NMD and other European networks could play a meaningful role in improving transition care. However, nearly one-third expressed uncertainty, reflecting either limited awareness or unmet expectations of what such networks can achieve in practice. This underscores the need for stronger communication and strategic engagement between ERNs and national health systems to support implementation of shared standards [11].

Our findings are consistent with previous research in other rare disease fields, which also demonstrate fragmented transition models, insufficient professional training, and limited integration of psychosocial care [1, 3, 4]. Our study adds to this literature by offering a comprehensive, multi-country perspective on transition practices specific to neuromuscular disorders, and by quantifying intra-country variability, which is often overlooked. Our findings also underscore the need to embed transition care within the policy for rare diseases, as advocated in rare disease policy initiatives such as the EU RD-ACTION [15].

Guidelines are currently lacking for the transition of young people with neuromuscular disorders (NMDs) to adult care, highlighting the need to develop consensus statements to guide this process. Our results and lessons learned from Duchenne muscular dystrophy (DMD) indicate that crucial elements of successful transition include starting the process early, involving patients and their families or carers, ensuring continuity of care at a comparable standard in adulthood as in childhood, and providing support that extends beyond healthcare needs [7].

## Conclusion

This survey reveals significant gaps and variability in the organisation and delivery of transition care for neuromuscular disorders. Although clinicians acknowledge the importance of structured, multidisciplinary transition processes, implementation is often inconsistent delayed, and poorly resourced, with limited collaboration beyond healthcare sectors. Key priorities include the clear communication of national policies, professional training, the establishment of frameworks in countries lacking them, and stronger coordination between paediatric and adult services.

To address these challenges, we recommend the development of a European roadmap for transition in NMDs, coordinated by EURO-NMD in collaboration with national stakeholders defining minimum quality standards, integrated care pathways, training modules for professionals, and adaptable implementation models. Additionally, reimbursement policies and institutional support for multidisciplinary transition clinics are essential to ensure equitable access to high-quality care.

Our survey highlights the underutilization of collaboration with other sectors such as schools, vocational rehabilitation services or local municipalities and their community health resources.

Future work should explore patient and caregiver perspectives, assess the impact of structured programmes where they exist, and promote harmonisation of transition practices to improve outcomes and ensure equitable, sustainable care for rare neuromuscular conditions.

## Supplementary Information

Below is the link to the electronic supplementary material.

Supplementary Material 1

Supplementary Material 2

## Data Availability

The datasets used and/or analysed during the current study are available from the corresponding author on reasonable request.
