# Supplementary material for: Transition from childhood to adulthood in neuromuscular disorders: results from the ERN EURO-NMD survey
Source: Orphanet J Rare Dis. 2025 Dec 9;21:15. doi: 10.1186/s13023-025-04144-x (PMC12801504; doi:10.1186/s13023-025-04144-x)
Supplement: Supplementary file 1 — Supplementary Material 1 [file 13023_2025_4144_MOESM1_ESM.pdf]

European Reference Network | Neuromuscular Diseases (ERN EURO-NMD)

Transition from childhood to adulthood in Neuromuscular Diseases

A questionnaire for healthcare providers in Europe to ascertain the current practices

Transition from paediatric to adult care in neuromuscular disorders (NMD) is a critical process that requires careful planning and support to ensure continuity of care and optimal outcomes for patients.

This questionnaire aims to gather insights from healthcare providers involved in the transition process to identify current practices, challenges, and opportunities for improvement in transitioning patients with neuromuscular disorders from paediatric to adult care in Europe.

We thank you for your collaboration. We will keep you informed about the global results of this survey.

GLOSSARY

Transition is defined as an "**active process that addresses the medical psychosocial and educational needs of adolescents as they prepare to move from child- to adult-centered healthcare**."

Transfer refers to the **physical change in location** of where healthcare is provided. **A one-time event** that occurs when a youth is transferred out

* O

Demographic Information

1. **What is your age range?** *

- ◯ 25-35 years old
- ◯ 35-45 years old
- ◯ 45-55 years old
- ◯ > 55 years old

2. **What is your gender?** *

- ◯ Woman
- ◯ Man
- ◯ Other
- ◯ Prefer not to say

3. **What is your specialty?** *

- ◯ Adult neurologist
- ◯ Child neurologist
- ◯ Other

4. Other specialty: please specify *

[ ]

5. Please select your country: *

Sélectionnez votre réponse

6. Please select your HCP/hospital (Austria): *

Sélectionnez votre réponse

7. Please select your HCP/hospital (Belgium): *

Sélectionnez votre réponse

8. Please select your HCP/hospital (Bulgaria): *

Sélectionnez votre réponse

9. Please select your HCP/hospital (Cyprus): *

Sélectionnez votre réponse

10. Please select your HCP/hospital (Czech Republic): *

Sélectionnez votre réponse

11. Please select your HCP/hospital (Denmark): *

Sélectionnez votre réponse

12. Please select your HCP/hospital (Estonia): *

Sélectionnez votre réponse

13. Please select your HCP/hospital (Finland): *

Sélectionnez votre réponse

14. Please select your HCP/hospital (France): *

Sélectionnez votre réponse

15. Please select your HCP/hospital (Germany): *

Sélectionnez votre réponse

16. Please select your HCP/hospital (Greece): *

Sélectionnez votre réponse

17. Please select your HCP/hospital (Hungary): *

Sélectionnez votre réponse

18. Please select your HCP/hospital (Ireland): *

Sélectionnez votre réponse

19. Please select your HCP/hospital (Italy): *

Sélectionnez votre réponse

20. Please select your HCP/hospital (Latvia): *

Sélectionnez votre réponse

21. Please select your HCP/hospital (Lithuania): *

Sélectionnez votre réponse

22. Please select your HCP/hospital (Luxembourg): *

Sélectionnez votre réponse

23. Please select your HCP/hospital (Malta): *

Sélectionnez votre réponse

24. Please select your HCP/hospital (Netherlands): *

Sélectionnez votre réponse

25. Please select your HCP/hospital (Norway): *

Sélectionnez votre réponse

26. Please select your HCP/hospital (Poland): *

Sélectionnez votre réponse

27. Please select your HCP/hospital (Portugal): *

Sélectionnez votre réponse

28. Please select your HCP/hospital (Slovenia): *

Sélectionnez votre réponse

29. Please select your HCP/hospital (Spain): *

Sélectionnez votre réponse

30. Please select your HCP/hospital (Sweden): *

Sélectionnez votre réponse

31. Please select your HCP/hospital (United Kingdom): *

Sélectionnez votre réponse

32. Other UK center: please specify *

33. **Please select your center type:** *

- ◯ Paediatric centre
- ◯ Adult centre
- ◯ Paediatric and adult centre

Your experience with transition of NMDs patients

GLOSSARY

TRANSITION, from childhood to adult care: Transition is defined as an "**active process that addresses the medical psychosocial and educational needs of adolescents as they prepare to move from child- to adult-centered healthcare**."

TRANSFER, from childhood to adult care: Transfer **refers to the physical change in location** of where healthcare is provided. **A one-time event** that occurs when a youth is transferred out of the child health system and into the adult care system

34. **How many years of experience do you have working with patients with NMD?** *

- ◯ < 5 years
- ◯ 5-10 years
- ◯ 10-20 years
- ◯ > 20 years

35. **Have you received any specific training on transitioning patients, especially those with NMDs, from pediatric to adult care?** *

- ◯ Yes
- ◯ No

36. Please write any relevant comment on the situation in your center and/or country regarding **training on transitioning patients**:

37. **Do you feel adequately prepared to support patients with neuromuscular disorders during the transition to adult care?** *

- ◯ Yes
- ◯ No

38. Please provide us with some comments explaining your answer to the previous question:

Transition of care in your center and country

39. Does your **HCP/hospital** have any transition programs, protocols or guidelines for the transition process **in general**? *

◯ Yes

◯ No

◯ I do not know

40. Please share a link to relevant information and resources here. Alternatively, you can send related documents by email to: coordination@ern-euro-nmd.eu

41. Does your **HCP/hospital** have any transition programs, protocols or guidelines for the transition process **for NMDs**? *

◯ Yes

◯ No

◯ I do not know

42. Please share a link to relevant information here, or alternatively send the documents by email to: coordination@ern-euro-nmd.eu

43. Does your **country** have any transition programs, protocols or guidelines for the transition process **in general**? *

- ◯ Yes
- ◯ No
- ◯ I do not know

44. Please share a link to relevant information here, or alternatively send the documents by email to: coordination@ern-euro-nmd.eu

45. Does your **country** have any transition programs, protocols or guidelines for the transition process **for NMDs**? *

- ◯ Yes
- ◯ No
- ◯ I do not know

46. Please share a link to relevant information here, or alternatively send the documents by email to: coordination@ern-euro-nmd.eu

Characterisation of the transition process

47. **At what age** do you start preparing young patients for adulthood and adequate transfer to adult services? *

- ◯ 12 years of age or earlier
- ◯ Between 13-14 years of age
- ◯ Between 15-16 years old
- ◯ Between 17-18 years old

48. **In your opinion,** what would be the ideal age range for the transition process to start? *

- ◯ 12 years of age or earlier
- ◯ Between 13-14 years of age
- ◯ Between 15-16 years old
- ◯ Between 17-18 years old

49. In your hospital, is there **an upper age limit** for transferring patients to an adult clinic? *

- ◯ Yes
- ◯ No
- ◯ I do not know

50. Please specify the upper age limit: *

51. **In your center, is the process of transfer from paediatric to adult care a one-off process?**
(Meaning there is no preparatory transition period before the age limit to switch to the adults clinic) *

- ◯ Yes
- ◯ No
- ◯ I do not know

52. **Are there regular meetings or joint appointments between paediatric and adult care teams to discuss transition plans for patients with NMDs?** *

- ◯ Yes
- ◯ No
- ◯ I do not know

53. Where do those meetings take place? *

- ◯ In paediatrics clinics
- ◯ In adult clinics
- ◯ Both adult and paediatric clinics

54. Are pediatric and adult professionals always involved?

- ◯ Yes, both with similar frequency
- ◯ Yes, but more frequently paediatricians (paediatric neurologists)
- ◯ Yes, but more frequently adult neurologists
- ◯ Never paediatricians (paediatric neurologists)
- ◯ Never adult neurologists

55. **In your centre, is a preparatory transition program available before the first consultation in the adult clinic?** *

- ◯ Yes
- ◯ No
- ◯ I do not know

56. Who coordinates this preparatory transition program?

- ◯ Paediatricians (paediatric neurologists)
- ◯ Adult neurologists
- ◯ Both
- ◯ Other

57. Other: please specify:

58. What does the preparatory transition program include? (e.g. Interdisciplinary standard operating procedures, Transition database, information for patients and parents...)

59. **What criteria do you use to assess readiness for transition?**
You can select more than one answer *

- [ ] Transition Readiness Assessment Questionnaire (TRAQ) 5.0
- [ ] California Healthy and Ready to Work (HRTW) Transition assessment tool: health care self-care
- [ ] Self-Management Skills Assessment Guide (SMSAG)
- [ ] TRxANSITION Scale
- [ ] Other: please specify below
- [ ] None

60. If you selected "Other", please specify:

61. **In your center, who advises parents/young adults with neuromuscular diseases about the transition from child to adult care?** *

- ◯ A designated transition coordinator
- ◯ The patient's paediatric neurologist
- ◯ The patient’s general practitioner
- ◯ Other

62. Other: please specify

63. **How do you ensure continuity of care and adequate transfer of patient information between healthcare providers?**
You can select more than one answer *

- ☐ The electronic patient health record is also available at the adult clinic
- ☐ A care coordinator or case manager oversees the transition process
- ☐ Standardized Transition Protocols and check lists are in use and transferred to the adult clinic before transition
- ☐ All relevant medical records, such as medical history, treatment plans, medication lists, and diagnostic test results, are transferred securely from the paediatric clinic to the adult clinic prior to the transfer
- ☐ Other: please specify below

64. If you selected "Other", please specify:

Patient and Family Support

65. **How do you address the psychosocial needs and concerns of patients and families during transition?** *

- [ ] Psychologist
- [ ] Social worker
- [ ] Youth worker
- [ ] Transition nurse
- [ ] Other

66. Other: please specify below

Barriers to Transition

67. **What are the common barriers or challenges faced in transitioning patients with NMDs from paediatric to adult care?** *

	Strongly agree	Agree	Neutral
Need for a multidisciplinary team	◯	◯	◯
Lack of communication between adult and paediatric teams	◯	◯	◯
Limited adult providers willing or able to accept the patients	◯	◯	◯
Difficulty in obtaining timely appointments in the adult system	◯	◯	◯
Difficulty in obtaining paediatric medical records	◯	◯	◯
Lack of adequate clinical settings for young adults with special needs in the adult system (e.g. autism, intellectual disability, behaviour disorders or those in wheelchairs, etc)	◯	◯	◯
Emotional attachment of paediatric care	◯	◯	◯

providers to their patients

68. **[illegible] other barriers that you think of that [illegible]sition process?**

Parents/caregivers preference to remain in paediatric clinic

Lack of financial support for transition programs

69. **[illegible] financial barriers from health authorities [illegible] transition process (e.g. certain services [illegible]therapy are no longer paid by the [illegible]h system or insurance companies for [illegible])?** *

Scarce education/training in transition

Quality of Transition Care

70. **Do you/your institution have mechanisms in place to assess the quality of transition care provided to patients with NMDs?** *

◯ Yes

◯ No

◯ I do not know

71. Yes: Please explain how the quality of transition care is assessed:

72. **Are there any metrics or indicators used to evaluate the effectiveness of the transition process?** *

◯ Yes

◯ No

◯ I do not know

73. **Does the paediatrician, paediatric neurologist/team receive any follow up information about transferred patients' visits in the adult clinic, at least in the year following the transition?** *

- ◯ Always
- ◯ Often
- ◯ Occasionally
- ◯ Never
- ◯ I do not know

Future Directions and Improvements

74. **What improvements or changes would you like to see in the transition process for patients with neuromuscular disorders?**

75. **To your knowledge, are there any innovative approaches or best practices from other institutions or countries that could be adopted to enhance transition care?**

76. **In your opinion, which of the following are needed for improving the transition process? Please rate from 1 (minor need) to 5 (maximum need):** *

	1	2	3
Obtaining financial support	◯	◯	◯
Joint consultations between adult and paediatric teams	◯	◯	◯
Implementation of national recommendation about adolescent's transition to adult care system	◯	◯	◯
Establishment of a network of specialized care, including physicians, who care for adolescent and young adults with NMDs	◯	◯	◯
A dedicated coordinator responsible for transition	◯	◯	◯
Introducing transition courses in the training programs for child and adult neurologists			
Including the patient's views and preferences to the planning of transition			

w

77. **[illegible] that the European networks, and in [illegible]O-NMD can facilitate the optimisation of [illegible] for patients with NMDs in Europe?** *

78. A transition protocol lain how? *

79. What do you believe are the bottlenecks?

80. How and who could facilitate the optimisation of care transition for NMD patients? (e.g. european bodies, european projects; national health systems, others)

Ce contenu n'a pas été créé ni n'est approuvé par Microsoft. Les données que vous soumettez sont envoyées au propriétaire du formulaire.

Microsoft Forms
