# Supplementary material for: Transition from childhood to adulthood in neuromuscular disorders: results from the ERN EURO-NMD survey
Source: Orphanet J Rare Dis. 2025 Dec 9;21:15. doi: 10.1186/s13023-025-04144-x (PMC12801504; doi:10.1186/s13023-025-04144-x)
Supplement: Supplementary file 2 — Supplementary Material 2 [file 13023_2025_4144_MOESM2_ESM.docx]

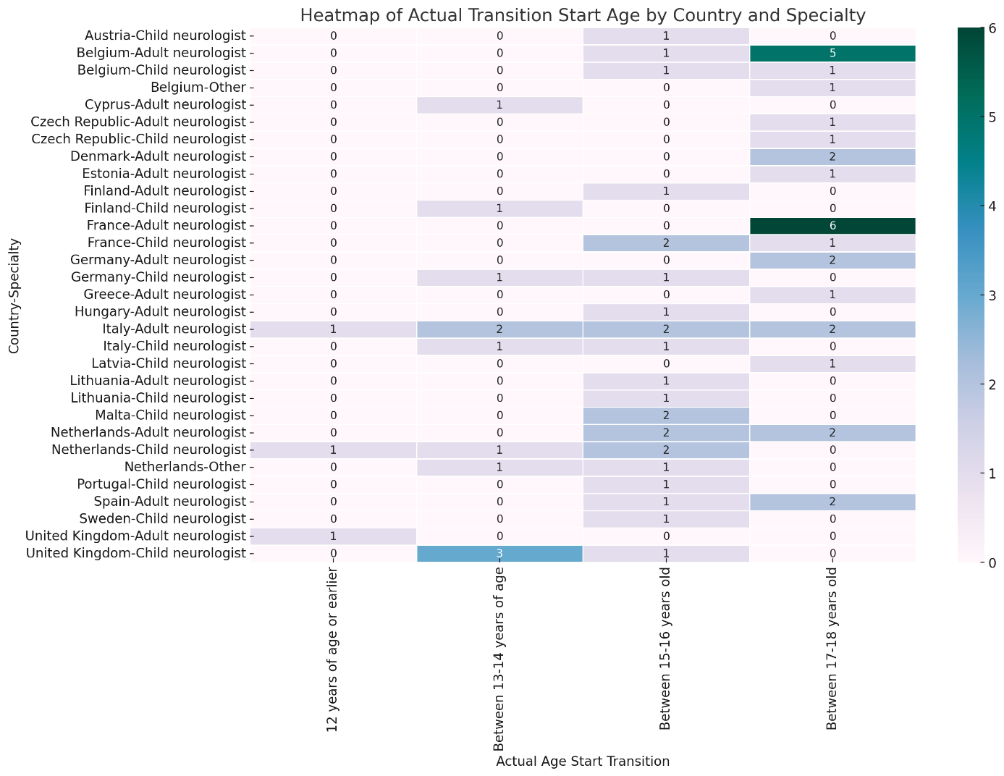


**Supplementary Figure 1:** Heatmap showing actual transition start age across different countries and specialties


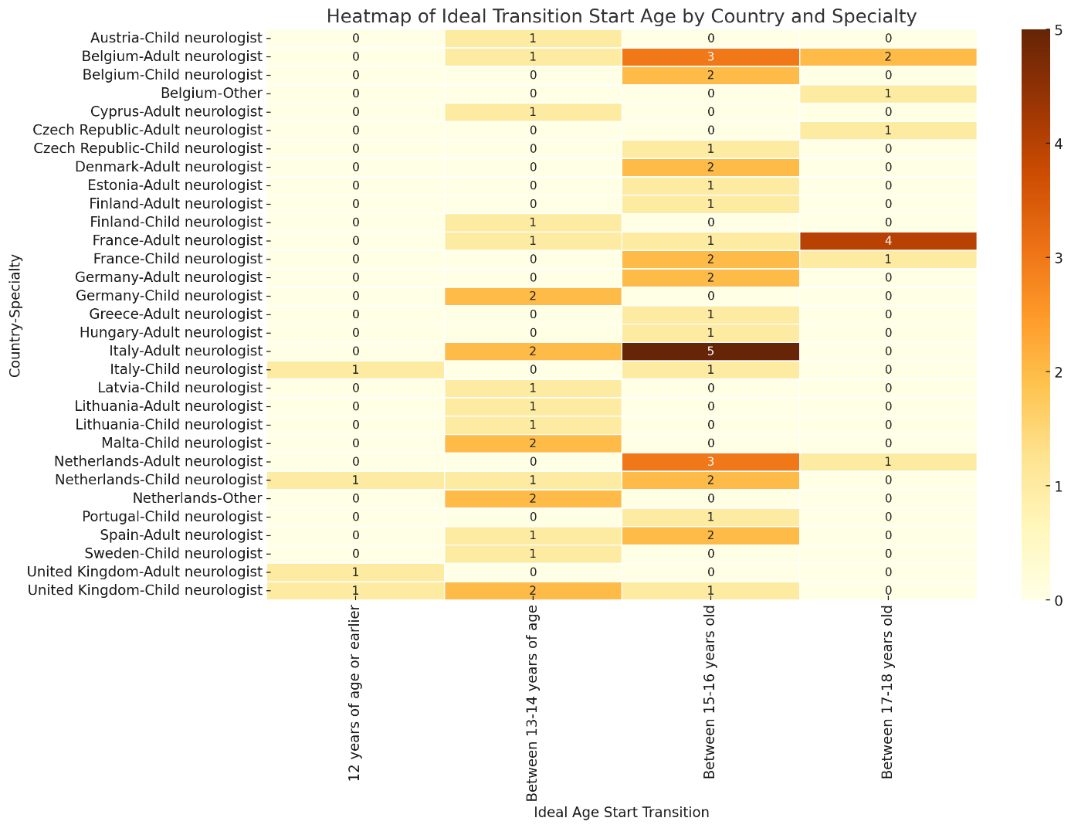


**Supplementary figure 2:** Heatmap showing ideal transition start age as perceived by healthcare professionals across different countries and specialties

**
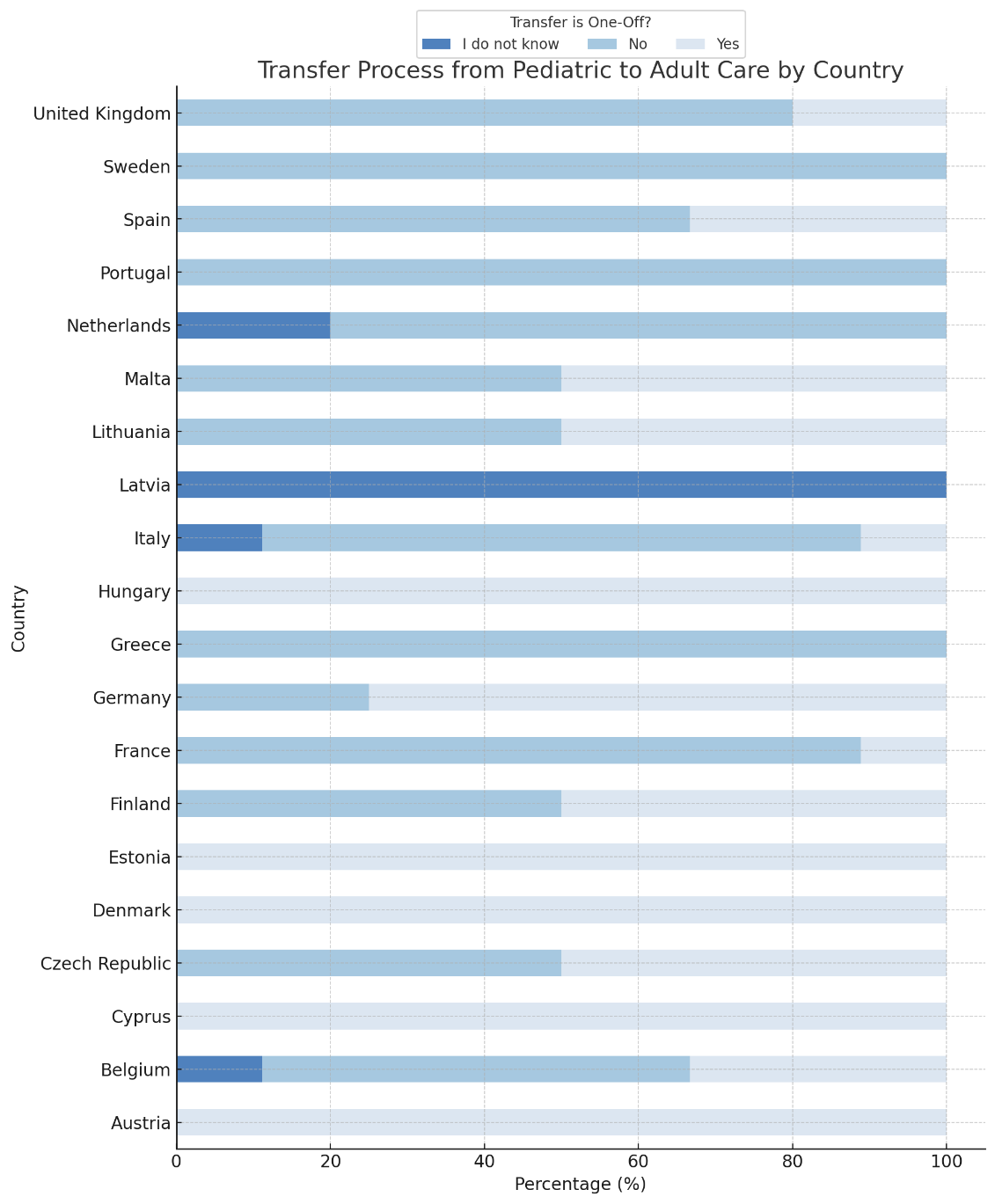
**

**Supplementary figure 3:** Bar chart representing the transfer process from paediatric to adult care per country.

**Supplementary file about barriers to transition**

**Full analysis of the qualitative responses**

#### 6.4 Additional Barriers Identified Through Open-Ended Responses

In response to an open-text question about additional barriers to transition, 25 healthcare professionals across 10 countries provided insights into challenges not covered by the predefined list. These responses were analyzed using a thematic content approach and grouped into five overarching themes.

**Time and Resource Constraints:** A recurrent theme was the lack of time and available resources to properly plan and implement the transition process. This was reported in Belgium, Denmark, France, Germany, and the UK. Clinicians noted that overloaded consultations, limited staff availability, and the absence of designated time for transition discussions impeded the process. Example (Belgium): *"Consultations are always very loaded/full... limiting the time that can be spent on talking about transition.".* Example (Germany): *"Only one appointment per year/half year... no extra time for transition."*

**Structural and System-Level Gaps:** Respondents from Italy, Portugal, Malta, and the Netherlands highlighted systemic challenges, including fragmentation between pediatric and adult care, lack of hub centers, and loss of supportive services in adult care. Some noted the rigidity of health system structures and poor integration of care across services. Example (Italy): *"The NHS is much better organized for children... supportive care initiatives are lost for adults.".* Example (Portugal): *"In adults, they have to come to the hospital on several days [instead of one]."*

**Legal, Policy, and Organizational Issues:** Contributors from Lithuania, Italy, and France mentioned broader policy and governance barriers. These included the absence of legal frameworks permitting adult specialists to consult minors, a lack of policy-maker engagement, and no dedicated funding or personnel to support transition. Example (Lithuania): *"There is no legal basis for adult specialists to consult minor patients, so they avoid it."* Example (Italy): *"Lack of knowledge and interest of policymakers about transition... no established funds."*

**Coordination and Communication Barriers:** Several HCPs across Belgium, France, Malta, and the Netherlands described coordination-related challenges such as difficulty organizing joint appointments, inadequate communication between pediatric and adult teams, and the ad hoc nature of transition meetings. Example (Malta): *"Transition meetings happen on an ad hoc basis... logistical issue."* Example (Netherlands): *"Making appointments for both specialists to be there at the same time can be challenging."*

**Attitudinal and Cultural Barriers:** Some responses pointed to cultural attitudes among clinicians and families that may hinder transition. These included perceived lack of prioritization by adult care providers and reluctance from parents to engage in transition planning. Example (Belgium*): "This does not seem to be a priority for adult physicians."* Example (Netherlands): *"Parents do not see the need to discuss this in mid-teen age... becomes an obstacle."*

The qualitative responses reinforced many of the previously ranked barriers but also revealed less tangible and often overlooked challenges such as logistical coordination, legal gaps, and cultural resistance. These findings highlight the importance of flexible, well-resourced systems that prioritize continuity of care, legal clarity, and interprofessional collaboration.

**Ranked Transition Barriers by Global Score**

| **Rank** | **Barrier** | **Mean Score** | **Interpretation** |
| --- | --- | --- | --- |
| 1 | Need for a multidisciplinary team | 1.9 | Agree |
| 2 | Scarce education/training in transition | 1.7 | Agree |
| 3 | Lack of financial support for transition programs | 1.6 | Agree |
| 4 | Lack of adequate adult clinical settings for young adults with special needs | 1.3 | Agree |
| 5 | Parents/caregivers prefer to remain in pediatric clinic | 0.8 | Agree |
| 6 | Emotional attachment of pediatric care providers | 0.6 | Neutral |
| 7 | Difficulty obtaining timely appointments in the adult system | 0.3 | Neutral |
| 8 | Lack of communication between pediatric and adult teams | 0.1 | Neutral |
| 9 | Limited adult providers willing/able to accept patients | -0.3 | Neutral |
| 10 | Difficulty obtaining pediatric medical records | -2.2 | Disagree |

**Supplementary file: Analysis of open-end responses regarding Financial Barriers to Transition**

The analysis of the open-ended responses revealed five recurring themes:

**1. Discontinuation or Reduction of Services after the age of 18 years**: Respondents from multiple countries indicated that essential services such as physiotherapy, psychological support, occupational therapy, speech therapy, and rehabilitation were no longer reimbursed or were markedly reduced once patients transition to adult care. This issue was reported in Austria, Estonia, Germany, Greece, Hungary, Italy, Latvia, Lithuania, Netherlands, Portugal, Spain, Sweden, and the UK. Examples: *"Physiotherapy or any other therapeutic/social worker next to the doctor are not paid in adult NMD medicine" (Germany). "In our country, physiotherapy is not covered by the state for adults" (Lithuania) "In adults, they have to come to the hospital on several days, unlike pediatric patients seen the same day" (Portugal)*

**2. Fragmented or Inadequate Funding Structures:** Respondents emphasized that while pediatric care is often well-structured and comprehensively covered, adult care lacks integrated funding mechanisms, particularly for multidisciplinary services. Some HCPs from Belgium, Czech Republic, Germany, Italy, and the Netherlands mentioned that financial support for adult care is either absent or not aligned with the complexity of NMD care. Examples: *"The process as a whole is not appropriately financed" (Germany). "No reimbursement code for joint visits or other transition consultations" (Italy). "Various care actions need to be repeated in adult clinics without extra payment" (Netherlands).*

**3. Age-Based Restrictions in Coverage:** Several HCPs noted age cut-offs imposed by insurance companies or national health systems, resulting in a sudden loss of services once patients turn 18. This was particularly prominent in Czech Republic, Lithuania, and the Netherlands. Examples: *"IvIg is reimbursed in myasthenic patients till 18 years but stopped after" (Lithuania). "Payment for pediatricians stops at the 18th birthday" (Netherlands)*

**4. Lack of Dedicated Budgets for Transition:** Some centers reported that transition-specific services—including case coordination, joint consultations, or preparatory programs—are not financed at all. This was noted in Belgium, Germany, and Italy. Examples: *"There is no dedicated budget for the transition process" (Belgium). "Personnel (case manager) to support the transition process is not foreseen" (Italy)*

**5. Broader Implications of Financial Barriers:** Beyond direct healthcare costs, some respondents, particularly from Italy and the UK, noted that indirect costs and social support gaps—such as transportation, work absences, or educational accommodations—are not covered during adulthood, adding further strain on patients and families. Examples: *"Financial support should be guaranteed for absences from work and to enhance therapy access" (Italy). "Young people are fearful of external carers; a transition program should help—but none exists" (UK)*
